# miR-6089 may prevent the inflammatory events leading to cardiovascular disorders in RA patients

**DOI:** 10.1016/j.heliyon.2024.e36763

**Published:** 2024-08-23

**Authors:** Afsaneh Shamsi, Seyed Askar Roghani, Mohammad Shamsi, Cyrus Jalili, Mahdi Taghadosi, Parviz Soufivand

**Affiliations:** a Immunology Department, Faculty of Medicine, Kermanshah University of Medical Sciences, Kermanshah, Iran; b Student Research Committee, Kermanshah University of Medical Sciences, Kermanshah, Iran; c Medical Biology Research Center, Health Technology Institute, Kermanshah University of Medical Sciences, Kermanshah, Iran; d Clinical Research Development Center, Imam Reza Hospital, Kermanshah University of Medical Sciences, Kermanshah, Iran; e School of Dentistry, AJA University of Medical Sciences, Tehran, Iran; f Cardiovascular Research Center, Health Technology Institute, Kermanshah University of Medical Sciences, Kermanshah, Iran

**Keywords:** Rheumatoid arthritis, Cardiovascular disease, LncRNA-HIX003209, miR-6089, Cardiovascular risk factors

## Abstract

**Background:**

Cardiovascular disease (CVD) is the most important comorbid condition in rheumatoid arthritis (RA) patients. Dysregulated expression of non-coding RNA families has a critical role in RA-associated inflammatory events, including cardiovascular manifestations. The long non-coding RNA (lncRNA)- HIX003209 has a role in RA associated inflammation. In the current study, we investigated the association of HIX003209 and its downstream microRNA, miR-6089, with various cardiovascular and inflammatory biomarkers in RA patients.

**Material and methods:**

60 RA patients, including 30 newly diagnosed and 30 on-treatment patients were recruited in this study, and 30 healthy people were selected as a control group. The gene expression of HIX003209, miR-6089, and CXCR3 were measured using Real-time PCR. The CVD risk was measured using Systematic Coronary Risk Evaluation (SCORE) and Framingham Risk Score (FRS).

**Results:**

The gene expression of LncRNA-HIX003209 was elevated significantly in newly-diagnosed compared to under-treatment and control groups (p < 0.05). The miR-6089 gene expression was elevated significantly in under-treatment RA patients group compared to control group (p < 0.001). There was a significant positive correlation between LncRNA-HIX003209 with CXCR3 gene expression (p < 0.01, r = 0.341). There was a significantly negative correlation between the gene expression of miR-6089 with DAS-28 (p < 0.05, r = −0.309), NT-proBNP plasma level (p = 0.039, r = −0.268), and CXCL9 plasma level (p < 0.001, r = −0.421).

**Conclusion:**

Regarding its anti-inflammatory effects, miR-6089 may play an important role in preventing the pathological events of cardiovascular disorders in RA patients, through its inhibitory effects on inflammatory chemokines, such as CXCL9, and NT-ProBNP. Higher expression of LncRNA-HIX003209 may disrupt the anti-inflammatory effect of miR-6089 in RA patients.

## 1 Introduction

Rheumatoid arthritis (RA) is an autoimmune disease entity with a systemic inflammatory condition, apart from progressive destruction of cartilage and joints, leading to prominent decreased life quality and expectancy associated with multiple coexisting conditions and critical extra-articular manifestations. Among these, cardiovascular disease (CVD) is the major contributor to the increased morbidity and deaths in RA patients and happens 1.5 times more compared to the healthy population [[1], [2], [3]].

It has been documented that the underlying pathophysiology of raised cardiovascular risk in the RA population is relevant to traditional cardiovascular risk factors (Age, gender, BMI, blood pressure, smoking, dyslipidemia, diabetes, and sedentariness), the effect of medication, disease activity, and above all, "chronic inflammation of RA [[4], [5], [6]]". Systemic inflammation shares common pathophysiological mechanisms in RA and CVD, for example, important shared pathological features such as increased expression of adhesion molecules and infiltration of inflammatory cells with the predominance of macrophages and TH1 cells have been observed between atherosclerotic plaques and inflamed rheumatoid synovium which are the hallmarks of persistent inflammation [7]. It is well recognized, elevation in various circulating chemokines and cytokines by inducing infiltration of inflammatory cells such as TH1 and MQ can contribute to the systemic inflammation that causes heightened risk of CVD in RA patients [8,9].

Furthermore, it is worth mentioning that chronic inflammation has the potential to affect the local microenvironment of cells and cause significant epigenetic modifications that lead to the accumulation of unfavorable changes in the cardiac and vascular systems [10]. Epigenetic modifications cause changes in DNA accessibility and regulate gene expression through mechanisms, including DNA methylation, histone protein modification, and non-coding RNA activity in the nucleus [11]. The non-coding RNA family, as a critical epigenetic modulator, is a type of RNA that typically does not encode proteins and includes the long non-coding RNA (lncRNA) (>200 nucleotides) and small non-coding RNA (<200 nucleotides) such as micro-RNA (miRNA) [[12], [13], [14]]. LncRNAs can function as competitive endogenous RNA (ceRNAs) to impede the normal biological functions of miRNAs through miRNA sponging and to regulate the expression of the miRNA [15]. MicroRNAs also play important roles in many biological processes, and generally by binding to complementary sequences on the target mRNA suppress the translation of the target protein [16].

Numerous evidence has shown the pivotal role of non-coding RNA in inflammation, autoimmunity, and cardiovascular diseases [17,18]. Growing evidence links number of dysregulated LncRNA expression with the inflammatory response in RA and CVD. For example, lncRNA HOTAIR is expressed differentially in RA and, through targeting the miR-106b-5p/Smad 7, axis inhibits cell proliferation, invasion, and migration of human fibroblast-like synoviocytes [19,20]. The study found that LncRNA MAIT expressed increasingly in synovium and myocardial tissues of collagen-induced arthritis (CIA) mice, which can lead to cardiovascular disease [[21], [22], [23]].

Accordingly, a previous study introduced a novel lncRNA named lncRNA-HIX003209 that significantly upregulated in peripheral blood mononuclear cells (PBMCs) of RA patients [24]. LncRNA-HIX003209 plays a role in the pathogenesis of RA by activation macrophages through the nuclear factor-κB (NF-κB) signaling pathway. This novel lncRNA promotes inflammation by inhibition miR-6089, which downregulate the expression of TLR4 through acting as competitive endogenous RNA (ceRNA) mechanism [25]. HIX003209 act as a molecular decoy for miR-6089, it capture miR-6089 and prevents its inhibitory effect on TLR4 expression. TLR4 exerts a pivotal role in the inflammatory pathway through activation of NF-κB, the critical inflammatory transcription factor, which induces the expression of different genes encoding for proinflammatory cytokines, including TNF-α, IL-6, which collectively play an indispensable role in RA pathogenesis [26,27].

In addition, elevated levels of HIX003209 expression have been observed in atherosclerotic coronary tissues compared to normal coronary artery samples. An increase in inflammatory mediators such as TNF-a leads to the upregulation of lncRNA-HIX003209 in VSMCs (vascular smooth muscle cells), which promotes cell proliferation, migration, and inflammatory mediators secretion such as IL-6, TNF-α, and IL-1β [28].

For the clarification of the possible role of this lncRNA signaling pathway molecules (LncRNA-HIX003209, miR-6089, and TLR4) in the development of CVD events in RA patients, at first, using Real-Time-PCR technique we evaluated the gene expression of LncRNA-HIX003209, miR-6089, and TLR4 in the peripheral blood of RA and control groups and then, investigated the association of traditional CVD risk factors (age, BMI, blood pressure, sex, FBS, HDL/LDL, TG, TC), the score of two CVD risk prediction algorithms (SCORE and FRS), and two well-known cardiac biomarkers (HS-CRP and NT-proBNP) with LncRNA-HIX003209/miR-6089 expression in RA patients.

Like the general population, RA individuals are at an increased risk of CVD due to conventional CV risk factors such as age, sex, BMI, blood pressure, smoking, hyperlipidemia, and diabetes mellitus. Detecting high-risk individuals who will benefit from CVD preventive efforts requires estimating total CVD risk. For this purpose, in the general population, many CVD prediction algorithms have been developed and the most well-known are SCORE and FRS which include traditional CV risk factors [6].

Assessing cardiac biomarkers is a quick and precise method for diagnosing heart disease. B-type natriuretic peptides (NT-proBNP) are commonly used as biomarkers for the diagnosis and screening of heart failure and cardiac dysfunction, primarily secreted by ventricular cardiac myocytes in response to pressure or tension [[29], [30], [31]]. Likewise, C-reactive protein (CRP) defined by high-sensitivity methods (HS-CRP) is a marker of systemic inflammation that predicts and assesses cardiovascular risk in clinical practice [32]. Several studies also suggest that CRP is an indicator of subclinical cardiovascular events in patients with RA and higher CRP levels are associated with a greater risk of cardiovascular disease in this group [33,34]. Accordingly, in our previous study, we measured plasma levels of NT-proBNP and HS-CRP using the ELISA technique and immunoturbidimetric assay, respectively, in the peripheral blood of RA and control groups and used the results in this study [35].

Also, recently we provided evidence that CXCL9 chemokine and its receptor CXCR3, which induces infiltration of TH1 and MQ to the inflammatory sites, have correlated with different variables of traditional CVD risk factors and cardiac biomarkers including NT-proBNP and HS-CRP in RA patients and may be considered inflammatory factors that contributes to the pathogenesis of CVD in RA patients [36,37]. Furthermore, considering the obvious role of CXCL9 chemokine as the most powerful contributor to age-related chronic inflammation (iAge) and cardiac and vascular dysfunction, and its possible role in the pathogenesis of CVD events in RA patients, in our previous study we measured the plasma level of CXCL9 and gene expression of CXCR3 in RA and control groups and at the following, in this study, we also evaluate the association between CXCL9/CXCR3 axis with LncRNA-HIX003209 and miR-6089 gene expression in the newly-diagnosed and under-treatment RA patients peripheral blood [36,38].

## 2 Material and methods

### 2.1 Study population

This cross-sectional control-matched study enrolled 60 patients with RA, divided into two groups: 30 newly diagnosed (Patients who were not recieved any medication) and 30 under-treatment patients who were referred to Imam Reza Hospital, Kermanshah University of Medical Sciences (KUMS), from April 2022 to October 2022, and 30 healthy subjects after matching for age and sex. Our study excluded participants who had a history of systemic rheumatic diseases other than RA, chronic diseases such as cardiovascular, pulmonary, kidney, liver, etc., autoimmune, inflammatory, metabolic, and infectious diseases, as well as pregnant women. RA diagnosis was made by expert rheumatologists using the classification criteria of the American College of Rheumatology and the European Alliance of Associations for Rheumatology 2010 (ACR/EULAR 2010). This study followed the Declaration of Helsinki and was conducted with approval from the Ethics Committee of KUMS (IR.KUMS.MED.REC.1401.017) and all participants have signed the informed consent. Table 1 shows demographic information and DMARD dosage and demographic information of participant, which we recruited in previous study [36].

**Table 1:** The DMARD dosage and the demographic information of groups study.

Variables	New case	On-treatment	Control	*P*-value
**Number**	30	30	30	
**Age (years)**	48.80 ± 13.01	49.67 ± 10.51	48.10 ± 12.07	
**Sex**	Male (n = 5) Female (n = 25)	Male (n = 5) Female (n = 25)	Male (n = 5) Female (n = 25)	
**TJC**	3.33 ± 3.50	0.23 ± 0.97		*P* < 0.0001
**SJC**	3.20 ± 3.46	0.23 ± 0.97		*P* < 0.0001
**DAS-28**	3.60 ± 1.16	2.33 ± 0.66		*P* < 0.001
**Smoking status**	Positive (6.7 %)(n = 2)	Positive (10 %) (n = 3)	Positive (n = 0)	
**Medication**				
**MTX**^**1**^**(%)**	0	100	0	
**HCQ**^**2**^**(%)**	0	100	0	
**PSL**^**3**^**(%)**	0	100	0	
**Other DMARDs**^**4**^	0	0	0	

Data are Mean ± SEM; 1-Methotrexate (7.5–25 mg per week), 2-Hydroxychloroquin (200 mg per day).

3-Prednisolone (5–10 mg per day). 4- Disease Modifying Anti-Rheumatic Drug, TJC: tender joint count, SJC: swollen joint count. 5- Anti-hypertensive drug.

### 2.2 Quantitative polymerase chain reaction (qPCR)

Total RNA was extracted from peripheral blood samples using an RNX PLUS kit (SinaClon, Tehran Iran) in accordance with the manufacturer's instructions. The RNA concentration and purity were assessed using a NanoDrop 2000 UV–Vis Spectrophotometer (Thermo Scientific, USA). Complementary DNA was synthesized via reverse transcription with a cDNA synthesis kit (PARSTOUS, Iran). Using online websites (UCSC, Oligocalc, and Oligoanalyzer) primers were designed and as follows: LncRNA-HIX003209 forward 5^/^- ACTGCTCGCCAGAACACTAC-3^/^ and reverse 5^/^- GGTGAGGTTGATCGGGGTTT-3^/^; miR-6089 forward 5^/^- CCCGGGCCCGGCGT-3^/^ and reverse 5^/^- CCCGCCCCGCCCCAC-3^/^; TLR4 forward 5^/^- TGGAAGTTGAACGAATGGAATGTG-3^/^ and reverse 5^/^- ACCAGAACTGCTACAACAGATACT-3^/^; GAPDH (housekeeping gene) forward 5^/^ GAAACCTGCCAAGTATGATG-3^/^ and reverse 5^/^ -AGGAAATGAGCTTGACAAAG-3^/^ (Table 2).

**Table 2:** Forward and reverse primers of target genes for real-time PCR amplification.

Gene name	Primer name	Nucleotide sequence
**LncRNA-HIX003209**	**Forward**	ACTGCTCGCCAGAACACTAC
	**Reverse**	GGTGAGGTTGATCGGGGTTT
**miR-6089**	**Forward**	CCCGGGCCCGGCGT
	**Reverse**	CCCGCCCCGCCCCAC
**TLR4**	**Forward**	TGGAAGTTGAACGAATGGAATGTG
	**Reverse**	ACCAGAACTGCTACAACAGATACT
**GAPDH**	**Forward**	GAAACCTGCCAAGTATGATG
	**Reverse**	AGGAAATGAGCTTGACAAAG

The Real-time PCR analysis was accomplished in in a total volume of 15 μl that consists 7.5 μl of the master mix of SYBR Green (Ampliqon), 1 μl of cDNA, 0.5 μl of each of the forward and reverse primers, and 5.5 μl sterilized distilled water. The PCR reactions were conducted on the Light cycler 96 (Roche Applied Science, Penzberg, Germany) using the universal thermal cycling as follows: 1. a pre-incubation cycle with a temperature of 95 °C for 30 s (s), 2. forty cycles of 2-step amplification including 95 °C for 5 s and 60 °C for 30 s; 3. a melting cycle of 95 °C for 5 s, 60 °C for 60 s, and 95 °C for 1 s; 4. a cooling cycle with a temperature of 50 °C for 30 s. The relative gene expression for each sample was estimated by the Pfaffl method ((Ratio=(E_target_)^ΔCt target (control-sample)^/(E_Ref_)^ΔCt Ref(control-sample)^) [39].

### 2.3 Enzyme-linked Immunosorbent Assay (ELISA)

The plasma levels of CXCL9 and NT-proBNP were assessed by human sandwich Enzyme-linked Immunosorbent Assay (ELISA) (ZellBio GmbH, Germany, Cat.NO: ZB-10049C-H9648), (ZellBio GmbH, Germany, Cat.NO: ZB-11239C-H9648) in our previous studies based on the protocols of the ELISA kit [35,36].

### 2.4 Immunoturbidimetric assay

Using ADVIA 1800 Clinical Chemistry System (Siemens, Germany) based on latex-enhanced immunoturbidimetric we evaluated the concentration of high sensitivity CRP (HS-CRP) in plasma samples, according to the manufacturer's instructions (assay range: 0.16–10 mg/L) in our previous study [35].

### 2.5 Measurement of fasting blood sugar (FBS) and lipid profile

Six milliliters of peripheral blood were collected after 12 h fast in ethylene diamine tetra-acetate (EDTA) tubes for assessment of fasting blood sugar (FBS), and lipid profile which conducted in our previous study [36]. Plasma Glucose was measured by the glucose oxidase-peroxidase method (Biosystems, Barcelona, Spain), the total cholesterol, HDL, and LDL cholesterol, as well as triglyceride, was determined via enzymatic reactions using commercial kits according to the manufacturer's instruction (Biosystem, Barcelona Spain), results were read using fully automated 7020 chemistry analyzer (Hitachi, Tokyo, Japan).

### 2.6 Systematic Coronary Risk Evaluation (SCORE) and Framingham Risk Score (FRS) Calculation

Our study population's CVD risks were calculated using SCORE and FRS algorithms that consider plasma lipid levels, blood pressure, smoking, age, and sex. The SCORE algorithm was developed in 2003 by 12 European cohorts to evaluate the 10-year risk of CVD mortality, including fatal myocardial infarction. The FRS was developed and internally validated in the USA population to predict the 10-year risk of cardiovascular disease (CVD), which includes stroke, peripheral artery disease, and heart failure [[40], [41], [42], [43]].

### 2.7 Disease activity score-28 (DAS-28) calculation

Using the formula (DAS28 = 0/56 + 0/28 (SJ)+0/70 In (ESR)+0/014 GH, (TJ: number of tender joints from 28 joints, SJ: number of swollen joints from 28 joints, GH: global health, ESR: erythrocyte sedimentation rate) DAS-28 was calculated.

### 2.8 Statistical analysis

SPSS software version 24.0 (SPSS, Chicago, IL, USA) and software GraphPad Prisms® 6.0(GraphPad Software, La Jolla, California, USA) were applied for the statistical analysis and graph drawing. The correlation between two variables was assessed using the Spearman and Pearson correlation and one-way ANOVA test was used to compare between three groups according to the normality distribution 1-sample Kolmogorov-Smirnov (K-S) test. P value marked as statistically significant at the level of <0.05.

## 3 Results

### 3.1 The plasma levels of FBS, lipids (LDL, HDL, TG, and cholesterol), HS-CRP, NT-proBNP, and CXCL9

The mean plasma concentration of FBS, LDL, HDL, TG, TC, HS-CRP, NT-proBNP, and CXCL9 in three groups which has been determined in previous study [36]are shown in Table 3.

**Table 3:** The mean plasma levels of FBS, TG, LDL, HDL, TC, HS-CRP, and NT-proBNP.

Variables	New cases (n = 30)	On-treatment (n = 30)	Control (n = 30)	*P*-value
**BMI (kg/m2)**	26.78 ± 5.01	24.76 ± 4.62	25.89 ± 3.69	0.223
**BpS (mm Hg)**	118.33 ± 20.52	115.33 ± 13.57	114 ± 15.44	0.593
**BpD (mm Hg)**	78 ± 7.14	80.66 ± 6.91	81 ± 4.02	0.191
**FBS (mg/dl)**	95.33 ± 16.08	91.44 ± 23.32	98.96 ± 17.37	0.093
**HDL (mg/dl)**	42.73 ± 10.67	57.86 ± 13.97	44.30 ± 8.82	<0.001
**LDL (mg/dl)**	93.03 ± 19.54	102.90 ± 22.76	92.03 ± 22.43	0.105
**TG (mg/dl)**	131.46 ± 59.19	112.60 ± 41.18	117.06 ± 29.13	0.241
**Cholesterol (mg/dl)**	169.96 ± 31.41	176.36 ± 39.77	165.36 ± 25.03	0.427
**FRS**	9.96 ± 11.02	7.20 ± 6.16	4.71 ± 6.07	0.029
**SCORE**	11.53 ± 12.19	8.83 ± 6.77	6.13 ± 6.60	0.078
**HS-CRP (mg/L)**	7.35 ± 6.82	3.33 ± 1.95	2.23 ± 0.62	<0.001
**NT-proBNP (Pg/ml)**	67.61 ± 12.47	61.43 ± 11.99	59.60 ± 10.69	0.016
**CXCL9 (ng/L)**	149.27 ± 35.11	127.54 ± 30.26	112.08 ± 21.66	<0.001

*Data are Mean ± SEM;* BMI: Body Mass Index, BPS: Systolic Blood Pressure, BPD: Diastolic Blood Pressure, FBS: Fasting Blood Sugar, HDL: High Density Lipoprotein, LDL: Low Density Lijpoprotein, TG: triglyceride, NT-proBNP: N-terminal pro–B-type natriuretic peptide, HS-CRP: High sensitivity C-reactive protein, CXCL9: Chemokine (C-X-C motif) ligand 9.

### 3.2 The comparison of variables in patients (new case + on-treatment) and control group

**Fig. 1:**
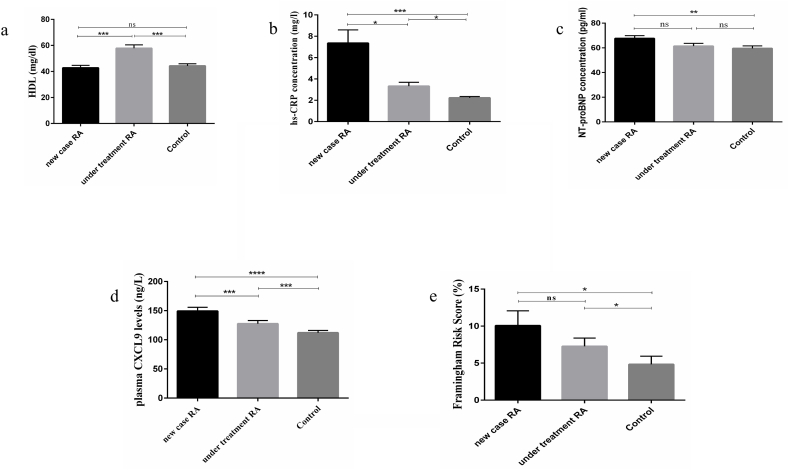
**Comparing the plasma levels of variables between three groups** Fig. 1**caption:** a) HDL was remarkably higher in under-treatment compared to newly diagnosed and control groups (*P* < 0.001 and *P* < 0.001). b) HS-CRP was significantly higher in newly diagnosed and under-treatment RA patients compared to the control group (*P* < 0.001 and *P* < 0.05) and was higher in the newly diagnosed group compared to the under-treatment group (*P* < 0.05). c) NT-proBNP plasma level was significantly higher in newly diagnosed compared to the control group (*P* < 0.01). d) The plasma level of CXCL9 significantly was higher in the newly diagnosed and under-treatment RA patients compared to healthy subjects (*P* < 0.0001 and *P* < 0.001, respectively). e) The CVD risk evaluated by the FRS algorithm was remarkably higher in newly diagnosed and under-treatment patients compared with healthy subjects (*p* < 0.05). (P < 0.05 = *, P < 0.01 = **, P < 0.001 = ***, P < 0.0001 = ****).

The mean FRS (p = 0.029) (Fig. 1e) and plasma levels of HDL (p < 0.001) (Fig. 1a), HS-CRP (p < 0.001) (Fig. 1b), NT-proBNP (p = 0.016) (Fig. 1c), and CXCL9 (p < 0.001) (Fig. 1d) were substantially different between the three groups.

### 3.3 The gene expression of CXCR3, LncRNA-HIX003209, miR-6089, and TLR4 in study groups

**Fig. 2:**
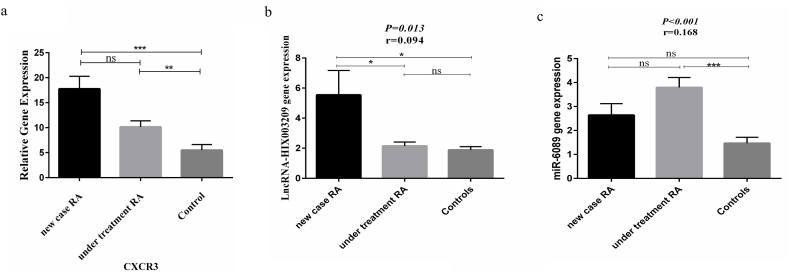
**Comparing the gene expression of CXCR3, LncRNA-HIX003209, miR-6089 among three groups** Fig. 2**caption:** a) The CXCR3 gene expression was significantly higher in newly diagnosed and under-treatment patients compared to healthy subjects (*P* < 0.001 and *P* < 0.01, respectively). b) The gene expression of LncRNA-HIX003209 was elevated significantly in newly-diagnosed compared to under-treatment and control groups (p < 0.05). The gene expression of miR-6089 was elevated significantly in under-treatment RA patients group compared to control group (p < 0.001). (P < 0.05 = *, P < 0.01 = **, P < 0.001 = ***, P < 0.0001 = ****).

The CXCR3 gene expression was significantly higher in newly diagnosed and under-treatment patients compared to healthy subjects (*P* < 0.001 and *P* < 0.01, respectively) (Fig. 2a).

The gene expression of LncRNA-HIX003209 was elevated significantly in newly-diagnosed compared to under-treatment and control groups (p < 0.05) (Fig. 2b).

The gene expression of miR-6089 was elevated significantly in under-treatment RA patients group compared to control group (p < 0.001) (Fig. 2c). Furthermore, there was no difference in TLR4 gene expression between the study groups.

### 3.4 Evaluation of correlation between variables in patients’ group (new case + on-treatment)

**Fig. 3:**
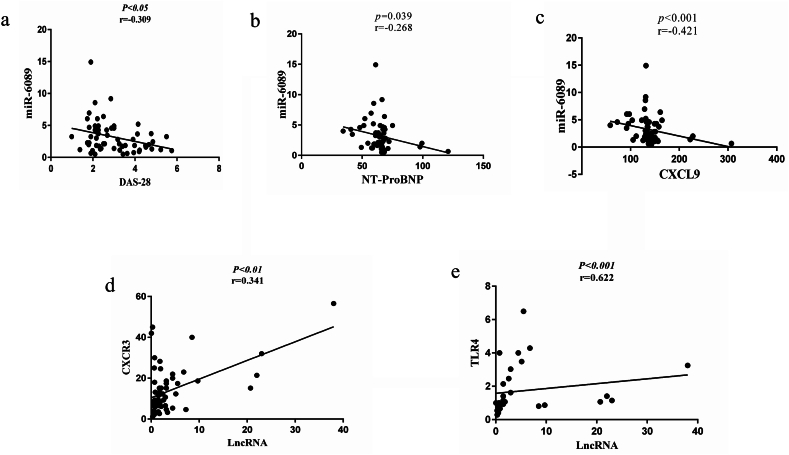
**Association between variables in patient groups (newly-diagnosed + under-treatment)** Fig. 3**caption:** a) There was a significantly negative correlation between the gene expression of miR-6089 with DAS-28 (p < 0.05, r = −0.309). b) There was a remarkable negative correlation between the gene expression of miR-6089 with NT-proBNP plasma level (p = 0.039, r = −0.268). c) There was a significant negative correlation between miR-6089 with CXCL9 plasma level (p < 0.001, r = −0.421). d) There was a significant positive correlation between LncRNA-HIX003209 with CXCR3 gene expression (p < 0.01, r = 0.341). e) There was a significant positive correlation between LncRNA-HIX003209 with TLR4 gene expression (p < 001, r = 0.622).

There was a significantly negative correlation between the gene expression of miR-6089 with DAS-28 (p < 0.05, r = −0.309) (Fig. 3a). There was a remarkable negative correlation between the gene expression of miR-6089 with NT-proBNP plasma level (p = 0.039, r = −0.268) (Fig. 3b). There was a significant negative correlation between miR-6089 with CXCL9 plasma level (p < 0.001, r = −0.421) (Fig. 3c). There was a significant positive correlation between LncRNA-HIX003209 with CXCR3 gene expression (p < 0.01, r = 0.341) (Fig. 3d). There was a significant positive correlation between LncRNA-HIX003209 with TLR4 gene expression (p < 001, r = 0.623) (Fig. 3e).

## 4 Discussion

Non-coding RNAs, including LncRNAs, have gained significant attention in medical research. They play crucial roles in the pathogenesis of various inflammatory diseases, including CVD and RA [21,44,45].

In this study, the expression of LncRNA-HIX003209 in the peripheral blood of RA patients was significantly higher in the newly-diagnosed group compared to the under-treatment and control groups (p < 0.05). Our findings align with a study conducted by Donghua Xu et al. that involved microarray screening and revealed that the expression of LncRNA-HIX003209 was higher in serum samples of RA patients. Furthermore, they found a positive correlation between LncRNA-HIX003209 expression and ESR, RF, and anti-CCP factors [46]. The levels of LncRNA-HIX003209 increase in response to inflammatory mediators. This increase promotes the proliferation and activation of classical macrophages, leading to an inflammatory pathway that produces NF-κB, this explains why newly diagnosed patients have higher levels of LncRNA-HIX003209 in their peripheral blood [47]. In our under-treatment group, the levels of the LncRNA-HIX003209 gene were probably affected by the use of anti-inflammatory and DMARDs drugs.

In current study, the relationship between TLR4 gene expression and LncRNA-HIX003209 in the peripheral blood cells of people with RA and the control group was investigated. We found a significant positive association between TLR4 gene expression and LncRNA-HIX003209 in the patient population (p < 0.001, r = 0.622), which indicates the enhancing effect of LncRNA-HIX003209/miR 6089 axis in inflammation caused by TLR4.

According to the study conducted by Xiaofeng Shi et al. inflammatory mediators such as ox-LDL, TNF-α, and PDGFBB increased the expression of HIX003209 in VSMCs. Increased aberrant expression of HIX003209 through downregulation of miR-6089 expression increases cell growth and migration and also induces the secretion of inflammatory mediators such as TNF-α, IL-6, and IL-1β in VSMC, which can eventually cause atherosclerotic plaques and thrombotic complications [28].

In this study for the clarification of possible role of LncRNA-HIX003209 in the development of CVD in RA patients, we evaluate the association of LncRNA-HIX003209 with various variable of CVD in the peripheral blood of RA patients.

We couldn't find any meaningful associations between LncRNA-HIX003209 gene expression with conventional CV risk factors and cardiac biomarkers, but interestingly, we found a significant positive correlation between LncRNA-HIX003209 with inflammatory chemokine receptors, CXCR3, in RA patients. Following the identification of the inflammatory chemokine CXCL9 as the strongest participant in the inflammatory ageing clock and in cardiac and vascular dysfunction, in our previous studies we showed the possible role of CXCL9 and its receptor, CXCR3, in the occurrence of cardiovascular disorders in rheumatoid arthritis patients [35,36,38]. Considering the existence of this significant positive correlation, it can be said that LncRNA-HIX003209 probably also plays a role in the pathogenesis of CVD in RA patients. In the following, to further clarify this issue, we also evaluated the target microRNA, miR-6089, of the LncRNA-HIX003209 gene.

It has been documented that LncRNA-HIX003209 acts as a competitive endogenous RNA in RA, and by binding and inhibiting miR-6089 through TLR4/NF-κB pathway in macrophages exaggerates inflammation [48]. We evaluated the gene expression of miR-6089 in the peripheral blood of both RA and control groups. The gene expression of miR-6089 was significantly higher in under-treatment RA group compared to control group (p < 0.001). miR-6089, as the target of the LncRNA-HIX003209 gene, by targeting and downregulating the TLR4 gene has anti-inflammatory properties, in parallel to our results, the higher expression level of miR-6089 gene in the under-treatment group compared to the control group is probably caused by the use of anti-inflammatory drugs. Previous documents showed that miR-6089 was significantly decreased in the serum exosome of patients with RA compared to healthy individuals, and on the other hand, with increased over-expression of miR-6089 followed by targeted control of TLR4 signaling, the production of IL-6, IL-29 and TNF-α cytokines decreases [49]. Interestingly, we found a significant reverse correlation between miR-6089 and RA clinical parameters, disease activity score-28 (DAS-28), in RA patients (p < 0.05, r = −0.309). Accordingly due to the anti-inflammatory property of miR-6089, as the inflammation and severity of disease activity increase, the amount of this microRNA decreases, which further confirms our obtained results.

Using the ELISA technique we evaluated NT-proBNP plasma levels in the peripheral blood of RA and control groups in our previous study [35]. Considering that NT-proBNP has been established as a gold standard biomarker in diagnosing heart failure and predicting early-stage heart abnormalities, in the current study, we evaluated the correlation between the miR-6089 with NT-proBNP. To the best of our knowledge, for the first time, we showed a significant reverse association between miR-6089 and NT-proBNP plasma levels in RA patients (p = 0.039, r = −0.268) which probably indicates the protective role of miR-6089 in the development of cardiovascular disorders in the RA population. Also, NT-proBNP is the well-established biomarker of left ventricular disorder which is prevalent CVD in the RA population [50,51]. This finding further supports the protective effect of miR-6089 in CVD.

Furthermore, investigating the relationship between the CXCL9/CXCR3 axis and miR-6089 showed promising results. For the first time, we found a significant reverse correlation between miR-6089 and CXCL9 plasma levels (p < 0.001, r = −0.261) in RA patients. CXCR3 and its ligands including CXCL9, CXCL10, and CXCL11, have a critical contribution in the pathogenesis of left ventricular disorder [52]. This finding also parallels the negative correlation between miR-6089 and NT-proBNP.

Considering that miR-6089 in RA patients had an inverse and significant correlation with DAS-28, NT-proBNP and CXCL9, it can be assumed that miR-6089 plays a role in the preventing the pathological events of cardiovascular disorders in RA patients. In addition, due to the significant positive correlation between LncRNA-HIX003209 and CXCR3, it can be said that LncRNA-HIX003209 probably also plays a pathological role in the development of cardiovascular disorders in RA patients. According to the previous studies, the increased expression of HIX003209 in atherosclerotic coronary tissues compared to normal coronary artery samples has been determined, it can be assumed that this lncRNA is probably related to other CVD risk factors in advanced stages of CV disorders and accumulation in atherosclerotic plaques.

## 5 Conclusion

Regarding its anti-inflammatory effects, miR-6089 may play an important role in preventing the pathological events of cardiovascular disorders in RA patients, through its inhibitory effects on inflammatory chemokines, such as CXCL9, and NT-ProBNP. Higher expression of LncRNA-HIX003209 may disrupt the anti-inflammatory effect of miR-6089 in RA patients.

## Funding

This work was supported by the 10.13039/501100005317Kermanshah University of Medical Sciences grant number (4,010,153).

## Data availability

The data generated and/or analyzed during the current study are available from the corresponding author on reasonable request.

## Ethics approval

This study was performed in line with the principles of the Declaration of Helsinki. Approval was granted by the Ethics Committee of 10.13039/501100005317Kermanshah University of Medical Sciences (Approval No: IR.KUMS.MED.REC.1401.017).

## Consent to participate

Informed consent was obtained from all individual participants included in the study.

## CRediT authorship contribution statement

**Afsaneh Shamsi:** Writing – review & editing, Writing – original draft, Methodology, Investigation, Data curation. **Seyed Askar Roghani:** Writing – review & editing, Software, Formal analysis. **Mohammad Shamsi:** Writing – review & editing, Writing – original draft, Investigation. **Cyrus Jalili:** Writing – review & editing, Formal analysis. **Mahdi Taghadosi:** Validation, Supervision, Project administration, Funding acquisition, Conceptualization. **Parviz Soufivand:** Writing – review & editing, Data curation.

## Declaration of competing interest

The authors declare that they have no known competing financial interests or personal relationships that could have appeared to influence the work reported in this paper.
